# A New Euler's Formula for DNA Polyhedra

**DOI:** 10.1371/journal.pone.0026308

**Published:** 2011-10-17

**Authors:** Guang Hu, Wen-Yuan Qiu, Arnout Ceulemans

**Affiliations:** 1 Department of Chemistry, State Key Laboratory of Applied Organic Chemistry, Lanzhou University, People's Republic of China; 2 Department of Chemistry and INPAC institute for Nanoscale Physics and Chemistry, Katholieke Universiteit Leuven, Leuven, Belgium; Università di Napoli Federico II, Italy

## Abstract

DNA polyhedra are cage-like architectures based on interlocked and interlinked DNA strands. We propose a formula which unites the basic features of these entangled structures. It is based on the transformation of the DNA polyhedral links into Seifert surfaces, which removes all knots. The numbers of components 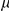, of crossings 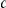, and of Seifert circles 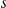 are related by a simple and elegant formula: 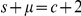. This formula connects the topological aspects of the DNA cage to the Euler characteristic of the underlying polyhedron. It implies that Seifert circles can be used as effective topological indices to describe polyhedral links. Our study demonstrates that, the new Euler's formula provides a theoretical framework for the stereo-chemistry of DNA polyhedra, which can characterize enzymatic transformations of DNA and be used to characterize and design novel cages with higher genus.

## Introduction

Polyhedral structures are basic markers of space, which have been known and celebrated for thousands of years [1]. They are encountered not only in art and architecture, but also in matter and many forms of life. The study of polyhedra has guided scientists to the discovery of spatial symmetry and geometry. A great theorem, which descends from geometry to topology, is Euler's polyhedral formula [2], [3]

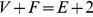
(1)where *V*, *F* and *E* are the respective total numbers of vertices, faces and edges of the polyhedron. Separate relations may also be established between pairs of these structural elements. As an example, let *n_i_* denote the degree of the *i*-th vertex, and let *p_j_* denote the number of sides to face *j*, with 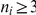 and 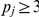. Then, we have:
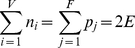
(2)In particular, for the regular polyhedron where *n* edges radiate from every vertex, and every face is a *p*-gon, this becomes
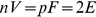
(3)Euler's formula also provides a simple way for characterizing symmetry properties of polyhedral molecules [4].

In recent years entirely new types of polyhedral molecules, based on DNA, have emerged [5], which challenge our ideas of what is possible in the chemical and biological world. Since the synthesis of the DNA cube [6], a rich variety of DNA polyhedra, including tetrahedron [7], octahedron [8], dodecahedron [9], icosahedrons [10], and buckyball [11] have now been reported in the literature. In these nano-constructions, each face is made of closed, interlocked DNA rings, each edge is made of double-helix [12] or quadruplex-helix [13]–[15] DNA strands, and each vertex represents an immobile multi-arm junction. The interest in these species is rapidly increasing not only for their potential properties but also for their intriguing architectures and topologies. The unresolved conflict has impelled a search for an even deeper understanding of nature.

To address these structural puzzles, we were led to the mathematical models of so-called polyhedral links [16]–[21], the rigorous mathematical definition of which was investigated by Jablan *et al* [22]. Polyhedral links are not simple, classical polyhedra, but consist of interlinked and interlocked structures, which require an extended understanding of traditional geometrical descriptors. Links, knots, helices, and holes replace the traditional structural relationships of vertices, faces and edges. The stereochemical control of these curious objects is still in its infancy, and would greatly benefit from clear theoretical models which express the relationships between the constituent descriptors, much in the same way as Euler's formula has done for the classical polyhedra. A challenge that is just now being addressed concerns how to ascertain and comprehend some of the mysterious characteristics of the DNA polyhedral folding. The needs of such a progress will spur the creation of better tools and better theories.

Our treatment is based on the standard apparatus of knot theory [23], [24]. A convenient way to facilitate the study of knots and links in terms of geometry makes use of the Seifert algorithm [25], [26], which provides a surprisingly simple connection between knots and links and 2*D* surfaces. Euler's polyhedral formula has already provided a powerful tool to study the geometry of classical and regular polyhedra. Our aim is to use the Seifert surface to find the new Euler's formula for some twisted and complex polyhedra, in view of revealing the intrinsic mathematical properties and controlling the supramolecular design of DNA polyhedra. The new Euler's formula would offer a novel and profound modification to our theoretical description of the geometrical and topological structures of the polyhedral links.

## Methods

Polyhedral links are mathematical models of DNA polyhedra, which regard DNA as a very thin string. More precisely, they are defined as follows.

### Definition 1. A polyhedral link L is an interlinked and interlocked architecture that is obtained form a polyhedral graph G, by using tangle structures to replace its vertices and edges

**Figure 1:**
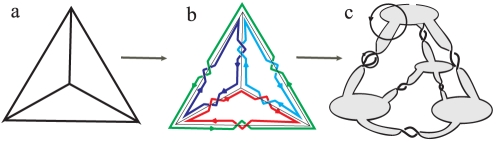
The Seifert construction. The construction of the *T*_2_-tetrahedral link from a tetrahedral graph and the construction of *Seifert surface* based on its minimal projection. Each strand is assigned by a different color. The Seifert circles distributed at vertices have opposite direction with the Seifert circles distributed at edges. The arrows indicate the 5′ - 3′ direction of the DNA backbone.

Our previous work [18], [19] demonstrated that polyhedral links used to describe DNA polyhedra are all *alternating links*, which contain crossings alternate between over and undercrossings along one component circuit. An example of a tetrahedral link is constructed from an underlying tetrahedral graph shown in Figure 1. The edges in this structure show two crossings, giving rise to one full twist of every edge. This example belongs to the class of *T*_2*k*_ polyhedral links [18], where *k* denotes the number of full-twists along each edge. In the present example *k* = 1. For the polyhedral graphs, the number of vertices, edges and faces, *V*, *E* and *F* are three fundamental geometrical parameters. In the context of knot theory, it is realized that crossing numbers *c*, component numbers *μ* and Seifert circle numbers *s* may be most three important invariants for polyhedral links.

### Definition 2. The crossing numbers c(L) of a polyhedral link L is the least number of crossings that occur in any projection of the polyhedral link

From this definition, a minimal graph of a polyhedral link with *c* crossing numbers is a projection that just has *c* crossings. It is easy to determine the minimal projection of an alternating link and our proofs are all based on minimal projections [19].

### Definition 3. The component number μ(L) of a polyhedral link L is the number of closed nonintersecting curves called components

**Figure 2:**
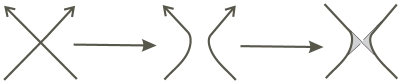
The operation of Seifert construction. The arrows indicate the orientation of the strands.

In the figures we always distinguish components by different colors. The definition of component number is more or less similar to the number of boundary components of thickened graphs, as defined in Ref [27]. Therefore, it is easy to compute *c* and *μ* by counting the crossings and component number in a minimal projection. For Seifert circle numbers, it needs applying Seifert algorithm, proposed by the German mathematician Herbert Seifert [28] in 1934, to generate the surfaces which have polyhedral links as boundaries also based on their minimal projections. In the case of DNA strands links are ‘oriented’, since DNA polymerization follows a direction of propagation from 5′ to 3′. This direction will be denoted by arrows. For links between oriented strips, the Seifert construction includes the following two steps (Figure 2):

1. Firstly, each link is ‘nullified’ by directly connecting tails and heads of intersecting arrows. In this way a set of nonintersecting circles called Seifert circles will be generated.
2. Secondly, these circles are again connected to each other at the position of the original crossing by twisted bands. In this way a Seifert surface is obtained with the link as boundary.

It is easy to show that every set of oriented links gives rise to an orientable Seifert surface, *i.e.* a surface with two sides which can be colored differently. Figure 1 illustrates the conversion of the tetrahedral polyhedron into a Seifert surface. Each disk at vertex belongs to the gray side of surface that corresponds to a Seifert circle. Six attached ribbons that cover the edges belong to the white side of surface, which correspond to six Seifert circles with the opposite direction. Due to the helix structure of DNA, intuitively, there are two kinds of holes in DNA polyhedra: the large “holes” located at vertices and the small “holes” at edges, thus Seifert circles are used to fill these kinds of “holes” during the Seifert construction.

### Definition 4. The Seifert circle number s(L) of a polyhedral link L is the number of Seifert circles distributed in an orientable surface with the polyhedral link as it only edge

So far two main types of DNA polyhedra have been realized. Type I refers to the simple *T_2k_* polyhedral links, as shown in Figure 1. Type II is a more complex structure, involving quadruplex links. In the following two sections, we perform the Seifert construction for both types, and obtain an elegant knot-theoretical equivalent of Euler's theorem for the case of DNA polyhedra.

## Results

### Type I polyhedral links

In their seminal paper, Chen and Seeman reported the first polyhedral catenane synthesized from DNA: the DNA cube [6]. Its edges consist of double-helical DNA with anti-orientation, and its vertices correspond to the branch points of the junctions. In our previous works [18], *T*_2*k*_ polyhedral links or branched polyhedral links have been constructed to describe the topology. In order to compute the number of Seifert circles, the minimal graph of a polyhedral link can be decomposed into two parts, namely, vertex and edge building blocks. Applying the Seifert construction to these building blocks of a polyhedral link, will create a surface that contains two sets of *Seifert circles*, based on vertices and on edges respectively.

**Figure 3:**
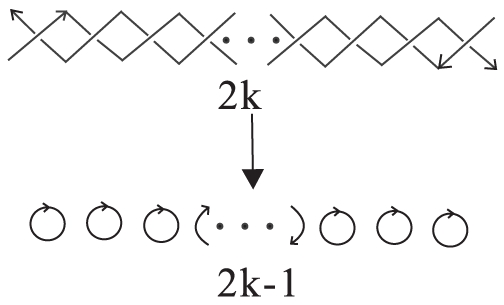
Annihilating 2*k* crossings generates 2*k*−1 Seifert circles.

As mentioned in the above section, each vertex gives rise to a disk. Thus, the number of Seifert circles 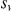 derived from vertices is:
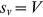
(4)where *V* denotes the vertex number of a polyhedron. An edge containing *k* anti-parallel full twists, as shown in Figure 3, presents 2*k* crossings which will generate 2*k*−1 circles.

So, the equation for calculating the number of Seifert circles 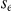 derived from edges is:
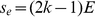
(5)where *E* denotes the edge number of a polyhedron.

Therefore, each half-turn on an edge and each central cavity of a vertex of a polyhedral link correspond to a Seifert circle. As a result, the number of Seifert circles 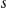 is given by:
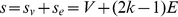
(6)Moreover, each edge is decorated with two turns of DNA, which makes each face corresponds to one cyclic strand. Thus, the number of closed DNA loops, called the component number *μ,* is given by:
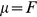
(7)where *F* denotes the face number of a polyhedron. In addition, the relation of crossing number *c* and edge number *E* is given by:
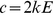
(8)The sum of Eq. (6) and Eq. (7) is:
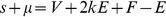
(9)Substitution of Eq. (8) into Eq. (9), yields:
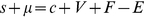
(10)Using Eq. (10) and Euler's formula for a polyhedron that can be mapped on a spherical surface of genus zero 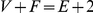, we can derive the following result:
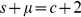
(11)When not all edges are of the same degree, the term *2kE* in the above derivation should of course be replaced by the more detailed form 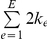, where *2k_e_* denotes the number of crossings on edge *e*, but this will not affect the further proof.

The formula (11) connects the number of Seifert circles *s*, and the component number *μ* and crossing number *c* of polyhedral links, in a way which is entirely analogous to Euler's famous formula. As a specific example of the Eq. (11), consider the DNA tetrahedron, characterized by: *V = 4*, *E = 6* and *F = 4*. For the tetrahedral link shown in Fig. 1, *k = *1, *μ = *4, then 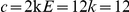 and *s* = *V*+(2*k*−1) *E* = 4+6×(2×1−1) = 10, thus 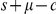 = 10+4−12 = 2 and of course Eq. (11) is always satisfied. It is easy to see that the number of Seifert circles is 10, with 4 located at vertices and 6 located at edges. In the DNA tetrahedron synthesized by Goodman *et al.* [7], four 30 base pair long oligonucleotides were appropriately designed to assemble six DNA duplex edges. As a result, each edge contains 20 base pairs that form two full-turns. In the related polyhedral link, *k* = 2, *μ* = 4, then 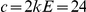 and 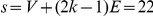, thus 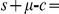
2 and Eq. (11) is also satisfied. Obviously, this new Euler's formula provides an elegant, consistent, and calculationally tractable framework for the DNA polyhedra.

### Type II polyhedral links

More recently, an alternative kind of DNA polyhedra with more complexity has also been synthesized [13]–[15]. First, *n* unique DNA single strands are designed to obtain symmetric *n*-point stars, and then these DNA star motifs were connected with each other by two anti-parallel DNA duplexes to get the final closed polyhedral structures. Accordingly, each vertex is an *n*-point star and each edge consists of two anti-parallel DNA duplexes. It is noteworthy that these DNA duplexes are linked together by a single-stranded DNA loop at each vertex, and a single-stranded DNA crossover at each edge. With this information we can extend our Euler formula to the second type of polyhedral links.

**Figure 4:**
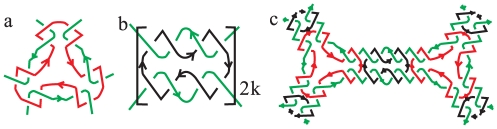
The building blocks of type II polyhedral links. (a) The vertex structure of a three-point star and (b) the edge structure of two anti-parallel DNA duplexes are connected to a star polyhedral link as shown in (c).

In type II polyhedral links, two different basic building blocks are also needed. First, we use ‘*n*-point star curves’ to replace the vertex of a polyhedron, where *n* is equal to the vertex degree. In general, 3-point star curves generate DNA tetrahedra, hexahedra, dodecahedra and buckyballs, 4-point star curves yield DNA octahedra, and 5-point star curves yield DNA icosahedra. The example of a 3-point star curve is shown in Figure 4(a). Then, we use ‘*m*-inverted twisted quadruplex-lines’, as shown in Figure 4(b), to replace the edge of a polyhedron. Each quadruplex-line contains a pair of double-lines, so the number of half-twists must be even, *i.e.*, *m* = 2*k*, where *k* denotes the integer number of full-twists on each edge. For the example shown in Figure 4, there are 1.5 turns of half-twists in each double-line, so *k* = 3 and the number of half-twists on each edge *m = 2k = 6*. Finally, these two structural elements are connected as shown in Figure 4(c). In view of their architectures, we call this type of polyhedral links ‘star polyhedral links’.

Although star polyhedral links exhibit more complex topological structures, the distribution of the Seifert circles is relatively simple when transforming into their Seifert surface. Here, we also consider vertices and edge building blocks based on minimal graphs, respectively, to compute the number of Seifert circles.

**Figure 5:**
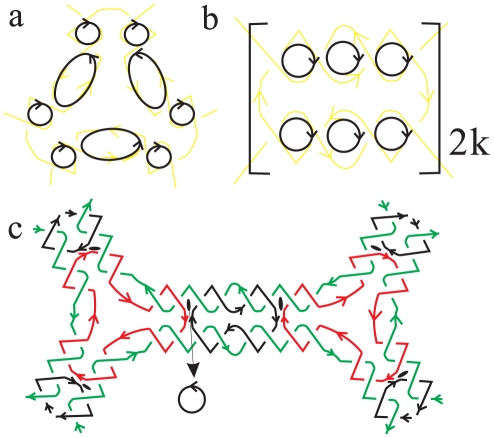
The distribution of Seifert circles. (a) Applying crossing nullifying to vertex building blocks, (b) Applying crossing nullifying to edge building blocks and (c) the distribution of Seifert circles at the connection between vertices and edges.

The application of crossing nullification to a vertex building block, corresponding to an *n*-point star, will yield 3*n* Seifert circles. As illustrated in Figure 5(a), one branch of 3-point star curves can generate three Seifert circles, so a 3-point star can yield nine Seifert circles. Accordingly, the number of Seifert circles 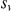 derived from vertices is:
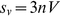
(12)By Eq. (3), we can further obtain:
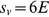
(13)Further application of crossing nullifying to an edge, consisting of 2*k*-inverted twisted quadruplex-lines, will generate *2k* Seifert circles, as shown in Figure 5(b). So, the number of Seifert circles 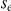 derived from edges is:
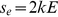
(14)Except for these Seifert circles obtained from vertices and edge building blocks, there are still additional circles which were left uncounted. In one star polyhedral link, there is a red loop in each vertex and a black loop in each edge. After the operation of crossing nullification, a Seifert circle appears in between these loops, which is indicated as a black bead in Figure 5(c). So the numbers of extra Seifert circles associated with the connection between vertices and edges is 2*E*.

For type II polyhedral links, therefore, the Seifert circles number 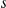 of their Seifert surface can be expressed as:
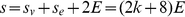
(15)Each face corresponds to a single-stranded DNA that forms duplexes, while each vertex and edge also contains a DNA single strand. For component number, the following relationship thus holds:
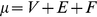
(16)In comparison with type I polyhedral links, crossings not only appear on edges but also on vertices. The equation for calculating the crossing number of edges 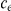 is:
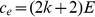
(17)and the crossing number of vertices 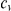 can be calculated by:
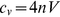
(18)Then, it also can be expressed by edge number as:
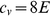
(19)So, the crossing number 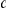 of type II polyhedral links amounts to:
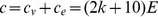
(20)Likewise, substitution of Eq. (1) and Eq. (20) into the sum of Eq. (15) and Eq. (16) gives the formula of Equation (11):
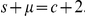
As an example consider the DNA icosahedron, with numbers of vertices, edges and faces *V* = 12, *E* = 30 and *F* = 20, respectively. For its synthesis, Zhang *et al.* [11] first designed twelve ‘5-point stars’ with unpaired loops of 5 base pairs as vertices of icosahedron. Any two adjacent vertices are connected by two parallel duplexes, with lengths of 42 base pairs or four turns. For the *k* = 4 polyhedral link mode based on the five-regular icosahedron, the number of Seifert circles located in vertices and edges are 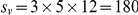, and 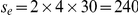, as shown in Figure 5(b), and at the connection between vertices and edges is 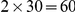, as shown in Figure 5(c). Therefore, the number of Seifert circles 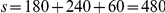, then we obtain 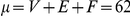 and *c* = (2*k*+10) *E* = 540, of course 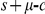 = 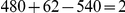 and Eq. (11) is established again. With the discovery of the new Euler's equation, fortunately, we now have a hope of solving some deeper mysteries.

## Discussion

### Validity and application

Equation (11) relates the Seifert circle number *s* of Seifert surfaces and the component number *μ* of links to the crossing number *c* of links. In comparison with the well known polyhedral Euler's formula 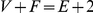, the formula 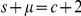 holds the same form. It is not difficult, intuitively at least, to see that the structural elements in the right-hand side of the equation have been changed from vertices and faces to Seifert circles and link components, and in the left-hand side from edges to crossings of helix structures. Accordingly, we state that the Eq. (11) is the “new Euler's Equation” for polyhedral links, with its Euler characteristic equals 2, which reveals the intrinsic property of DNA polyhedra. The classical “Euler's Equation”is the study of geometric properties of rigid objects, while the “new Euler's Equation” is detecting the topological characteristics of polyhedral links, such as connectedness, holes, and twistedness. Conversely, in formal, if retaining the number of vertices, faces and edges in Eq. (11), *i.e. s* = *V*, *mu* = *F*, *c* = *E*, will turn the new “Euler's Equation” back to the classical “Euler's Equation” and find where are the vertices, edges, and faces on a topological surface. The new Euler's equation pursues an inner harmony, where elegance, uniqueness and beauty define DNA polyhedra.

**Figure 6:**
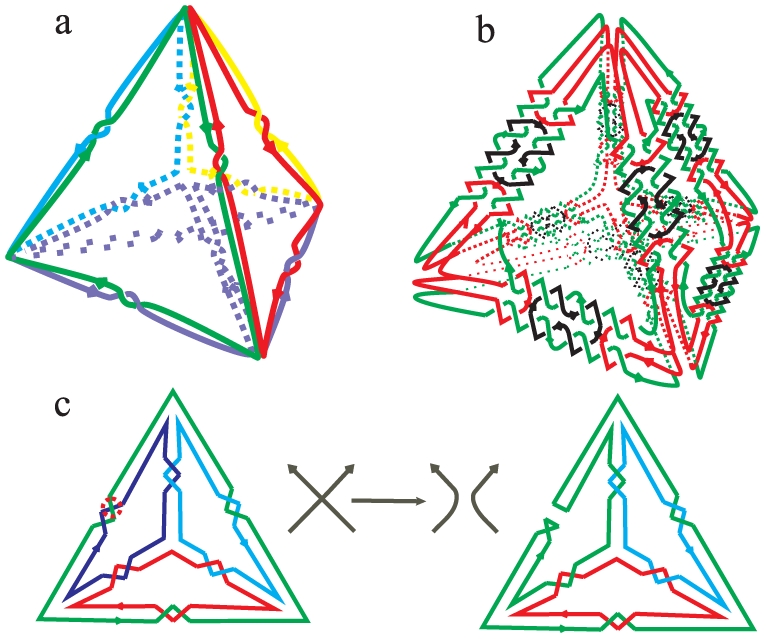
Two applications of the new Euler's formula. The type I (a) and type II (b) genus-one DNA polyhedra based on *K*_5_ graph. (c) The recombination of a tetrahedral link.

For a Seifert surface, there exist many topological invariants that can be used to describe its geometrical and topological characters. Among them, genus *g* and Seifert circle numbers *s* appear to be of particular importance for our purpose. Genus is the basic topological feature of a surface, which denotes the number of holes going through the surface. Euler's formula, as a geometrical property, can generalize to polyhedral nets on surfaces with other topologies as:
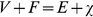
(21)Here 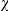 denotes the Euler characteristic, which is an invariant also, can be related to genus *g* by:
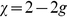
(22)As such, the Euler formula can be generalized to polyhedral links based on non-planar graphs:
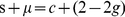
(23)As mentioned in the Euler formula (11) of branch and star polyhedral links, the Euler characteristic is 2, so we can conclude that the genus of a DNA polyhedron equals zero. The result shows that all DNA polyhedral catenanes synthesized so far are restricted to a surface homeomorphic to a sphere. The exciting implication is that genus is not only a purely mathematical definition, but also provides a heuristic principle for novel structures [29]. This opens the way to the design of novel polyhedral modes with *g*>0 for materials and DNA [30]–[32]. Jonoska and Twarock [32] have investigated all possibilities of constructing dodecahedral DNA cages theoretically. Using Eq. (23), it is easy to calculate that they are embedded on surfaces with genus 3, 4, 5 and 6, which builds a rich treasure house for chemical investigation. As examples shown in Figure 6(a) and (b), we propose another way to design two novel DNA polyhedra with *g* = 1, based on the complete graph of five nodes, *K*_5_, which is embedded on a torus. For the *K*_5_ graph, 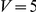, = 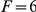, and 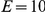. For its corresponding link shown in Fig. 6(a), the crossing number 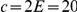, the component number 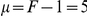 and the number of Seifert circles 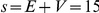. For its corresponding link shown in Fig. 6(b), the crossing number 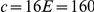, the component number 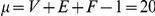 and the number of Seifert circles 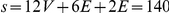. Hence, for both types of polyhedral links based on *K*_5_ graph, the new Euler formula satisfy 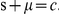


By analogy with the classic Euler's formula, we expect that such a simple and elegant relation will greatly contribute to an understanding of the topologically complex structures of polyhedral links, as well as some potential biological processes. As an example, we illustrate its use to characterize the recombinase regulation and controlling mechanisms for DNA polyhedra [33]. Recombinase is a site-specific enzyme, which, by cutting two segments and interchanging the ends of DNA, can result in the inversion or the deletion or insertion of a DNA segment. Thus, this operation exactly matches the crossing nullification in Seifert's algorithm! It means that the number of Seifert circles remains unchanged during the recombination, *i.e.* 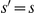. As shown in Figure 6(c), the recombination of a tetrahedral link changes the crossing number *c* by one, *i.e.*, 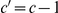. By inserting *c′* and *s′* into Eq. (11), one obtains 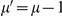. This means that each recombination will change the component number of polyhedral links by one, which proves Jonoska's result [27] of the change of DNA strands with regard to topological graph theory.

In knot theory, the crossing number serves as the basis for classifying knots and links. As an invariant, however, it is not very informative since different knots may have the same crossing number. Here, we propose that the Seifert circle number gives us a more satisfactory way to measure the complexity of polyhedral links. Rearranging Eq. (11) gives:
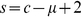
(24)As we can see from Eq. (24), Seifert circle numbers *s* consider not only crossing numbers *c* but also the additional information of component numbers *μ*. Such a modified descriptor is shown to be more effective than the crossing number *c*. Although this invariant is still not exclusive, it is an easily derived topological descriptor for DNA polyhedra. In DNA nanotechnology, crossing number *c* and component number *μ* are two experimentally accessible quantities. Crossing number *c* determined by the base number of DNA duplexes: *c*≈base number /5, and component number *μ* equals the number of circular DNA strands (DNA loops).

### Conclusions

In this paper, we have derived an intuitively simple formula for two types of polyhedral links by means of Seifert's algorithm. The new formula unites one geometrical invariant of the number of Seifert circles, and two knot invariants including crossing numbers and component numbers, in a single expression which is reminiscent of Euler's polyhedral formula. Furthermore, the study of two molecular descriptors, genus and Seifert circle number, may provide a new understanding of the structure of polyhedral links.

In conclusion, we believe that this research constitutes two main advances:

It provides a clear connection between the geometry of the underlying polyhedron and the knot structure of the entangled polyhedral links, which proves that each DNA polyhedra have their “own” Euler's formula.

It offers rigorous descriptors to quantify the geometry and topology of DNA polyhedra, and paves the way to the design of intrinsically novel structures.

This discovery could be instrumental to relate the toolbox of mathematical knot theory to biomolecular recombination processes, linking topology to reality-as Euler's polyhedral formula has done over and over again in the polyhedral world of molecules. The new Euler's equation would mark a beginning, not an end; it can be extended to objects with higher genus.
